# Mutations in Structural Genes of the Mitochondrial Complex IV May Influence Breast Cancer

**DOI:** 10.3390/genes14071465

**Published:** 2023-07-18

**Authors:** Ricardo Cunha de Oliveira, Sávio Pinho dos Reis, Giovanna C. Cavalcante

**Affiliations:** 1 Laboratory of Human and Medical Genetics, Graduate Program in Genetics and Molecular Biology, Federal University of Pará, Belém 66075-110, Brazil; oliveira.ca.ricardo@gmail.com; 2 Center for Biological and Health Sciences, State University of Pará, Belém 66087-662, Brazil; savio.reis@uepa.br

**Keywords:** breast cancer, mitochondria, oxidative phosphorylation, complex IV

## Abstract

Although it has gained more attention in recent years, the relationship between breast cancer (BC) and mitochondrial oxidative phosphorylation (OXPHOS) is still not well understood. Importantly, Complex IV or Cytochrome C Oxidase (COX) of OXPHOS is one of the key players in mitochondrial balance. An in silico investigation of mutations in structural genes of Complex IV was conducted in BC, comprising 2107 samples. Our findings show four variants (rs267606614, rs753969142, rs199476128 and rs267606884) with significant pathogenic potential. Moreover, we highlight nine genes (*MT-CO1*, *MT-CO2*, *MT-CO3*, *CO4I2*, *COX5A*, *COX5B*, *COX6A2*, *COX6C* and *COX7B2*) with a potential impact on BC.

## 1. Introduction

Breast cancer (BC) is the malignant neoplasm that most often leads women to death worldwide, affecting diverse socioeconomic classes. In 2020, the Global Cancer Observatory (GLOBOCAN) estimated 2,261,41 new cases and 684,996 deaths due to BC, a relevant growth compared to previous years [1,2,3,4]. Mutations in mitochondrial (mtDNA) and nuclear (nDNA) genes may influence this growth, being linked to several biological processes, such as energy generation through oxidative phosphorylation (OXPHOS), which is dysregulated in tumor progression [5,6].

Tumor progression can lead to metastasis and become the main cause of death in patients with BC, making the prognosis even more complex, since there is an increase in the ability to invade and establish in other tissues [7]. Among the alterations that the neoplastic cell performs for metastasis, there is the dysregulation in the mitochondrial energy production, called the Warburg Effect, in which OXPHOS has decreased activity, and glycolysis takes over the main role in energy production [8,9]. This phenomenon is postulated as a hallmark of many types of cancer, being linked to factors, such as mtDNA mutations [10]. However, BC is a type of cancer that may not usually follow this effect, indicating the need to study factors intrinsic to cellular metabolism such as mitochondrial OXPHOS in humans [11,12].

Cellular energy is related to mitochondria, which have key functions, such as control of apoptosis, control of oxidative stress, hydroelectrolytic balance and others [13]. Mitochondria have a heterogeneous and complex role in all types of cancer, highlighting the need for their study in BC [14]. Instability of cellular energy mechanisms may favor the malignant evolution of the disease, which is related to a worse prognosis and metastasis as it can lead to oxidative stress (linked to excessive production of reactive oxygen species—ROS), resistance to apoptosis and, consequently, increased tumor invasion.

This reinforces the multifactorial character of BC, where not only nuclear genetic instability can influence the progressive tumor cascade but also other cell organelles, such as mitochondria, which can indicate a cancerous cascade and influence other important genes such as oncogenes. It corroborates the fact that the mitochondrial genome can undergo many changes due to where it is located, mitochondrial cristae and direct contact with ROS, and COX is responsible for part of the elimination of these residues, making its presence essential, both for mitochondria and for the cell [5,15,16,17,18].

One of the main structures related to the control of mitochondrial oxidative stress is Complex IV, also known as Cytochrome C Oxidase (COX), present at the end of the electron transport chain (ETC). COX gains special importance to other types of complexes for being linked to the hydroelectrolytic balance and stabilization of the mitochondrial membrane potential. It is also COX that controls the rate limit of OXPHOS, being the only ETC complex that does not produce ROS [19,20]. In humans, COX is composed of 14 protein subunits, of which 3 are mitochondrially encoded and considered the main subunits (COX1, COX2 and COX3), and the other 11 structural subunits are encoded by nDNA (protein structures COX4, COX5A, COX5B, COX6A, COX6B, COX6C, COX7A, COX7B, COX7C, COX8 and NDUFA4 or COXFA4), reinforcing the fact that constant interaction occurs between mitochondria and nucleus, which can emphasize that genetic destabilization of COX and consequent change in its structure can lead to the progression of BC [21].

Therefore, COX is an essential constituent of OXPHOS, for which there is still no clear description in BC when compared to the other complexes (I, II and III) that have already been listed as closely related to the production of ROS (oxidative stress), metastasis and being considered as possible biomarkers for enabling the presence of oncogenic pathways in BC and other types of cancer, although more recent studies demonstrate that its destabilization promotes a cascade of aggressive proliferation and it is overexpressed in metastatic tumors; therefore, due to the lack of understanding and characterization of COX in breast cancer, there is an urgent need for studies that can help in its influence in this type of cancer, since alterations in other complexes are already associated with BC [12,21,22,23]. These findings reinforce the need to search for variants in the mitochondrial and nuclear genes that constitute COX with the potential to influence the progress of BC. Here, we investigated the presence of variants in genes related to the structure of Complex IV of OXPHOS in BC samples from public databases (DBs) to suggest potential genetic markers for this disease.

## 2. Materials and Methods

### 2.1. Characterization of the Study

An in silico, descriptive and exploratory study was carried out with data present in publicly available databases using open-source software. As the study was conducted with genomic sequences from public databases without individual identification and did not include new biological samples from patients, there was no need for submission or approval of this research to the Research Ethics Committee.

### 2.2. Study Design

The study was divided into Phase 1 (P1), in which the mitochondrial genes were analyzed, and Phase 2 (P2), for the analysis of the nuclear genes, totaling 20 structural genes. Mitochondrial genes were selected based on the current literature, and the nuclear genes were filtered according to mitoXplorer platform [24]. Table 1 shows the genes that were included in this study.

### 2.3. Sampling from Public Databases

#### 2.3.1. Analysis of Variants in Mitochondrial Genes (P1)

Phase 1 of this research was carried out with The Cancer Mitochondria Atlas (TCMA), available at https://ibl.mdanderson.org/tcma/about.html, accessed on 29 July 2022 [29]. Both SNP (Single-Nucleotide Polymorphism) and INDEL (Insertion/Deletion) types were investigated in the mitochondrial genes *MT-CO1*, *MT-CO2* and *MT-CO3*, in 216 BC samples [29]. TCMA does not have secondary characteristics, such as the clinical profile of the patients, so these were not analyzed in the P1 approach. Only the histological subtype was present in this DB, but because of the lack of more information, it was not analyzed.

#### 2.3.2. Analysis of Variants in Nuclear Genes (P2)

For the analysis of variants in the 11 nuclear Complex IV genes, we employed the public platform cBioPortal for Cancer Genomics, available at https://www.cbioportal.org/, accessed on 29 July 2022 [30,31]. Only DBs of BC patients were included, totaling 1891 BC samples from breast tumor and blood, and the analysis of clinical features was conducted considering different variables: age at diagnosis, sex and tumor subtype/classification.

### 2.4. Selected Samples

The selected DBs are characterized in Table 2, totaling eight DBs and 2107 samples.

### 2.5. In Silico Association and Functional Enrichment

The found variants were used to create the association network between the DBs. For this, R language [52] was employed, in addition to Microsoft Excel for the tabulation of these mutations. Genes that had more mutations among the DBs, as well as genes with variants of significant pathogenicity, were taken for functional enrichment analysis in Kyoto Encyclopedia of Genes and Genomes (KEGG) [53], Gene Ontology (GO) [54,55] and GeneMANIA [56]. Afterwards, the mutations were characterized using SNPNexus [57].

### 2.6. Statistical Analyses

In this work, JASP software and R language were used for the identification of mutations in the investigated COX genes, in addition to the use of the chi-squared test to verify the distribution of the data presented in the DBs [53,58]. The variables were in accordance with the molecular classification proposed by Dai and collaborators and the American Joint Committee on Cancer (AJCC) [59,60]. Histological classification was in accordance with Nottingham Histologic Score System and the pattern of metastasis with the Union for International Cancer Control (UICC). *p*-values < 0.05 were considered statistically significant. The metastasis staging is indicated by the presence of distant metastasis (M), each letter being followed by a number referring to the degree in which the malignant neoplasm is found, with M0 indicating that there is no distant metastasis, M1 that there is distant tissue metastasis and MX that it is not possible to access this information or that it is not clear whether, finally, CM0+ indicates that there is circulation of tumor cells in the blood or other regions of the body that are not the tumor; similarly, cancer can also be classified into stages I-IV according to its clinical evolution considering the presence or absence of metastasis.

### 2.7. Pathogenicity of Characterized Variants

To investigate the potential to cause damage and to be related to unbalanced pathways, three online platforms were used to assess the pathogenicity of the mutations and the possible effect according to their presence: SIFT (Sorting Intolerant From Tolerant); PolyPhen-2 (Polymorphism Phenotyping v2); and ClinVar [61,62,63].

## 3. Results and Discussion

### 3.1. Presence of Alterations in Mitochondrial Genes

From the total alterations, 169 unique variants were identified from the mitochondrial and nuclear databases. To the best of our knowledge, none of the nuclear genes’ variants were reported in some sort of bibliography until this work. Regarding mitochondrial variants, from a total of 137 alterations, 124 unique variants were identified in the three genes. Of these 124 alterations, 58.1% (n = 72) had not yet been reported in dbSNP and, regarding the types of mutations, 4.1% (n = 5) were frameshift, 15.3% (n = 19) were silent and 80.6% (n = 100) were missense or nonsense. Supplementary Table S1 shows all alterations found in the DB investigated for mtDNA.

Interestingly, 77 different alterations (62.1%) were found in *MT-CO1*, 24 (19.4%) in *MT-CO2* and 23 (18.5%) in *MT-CO3*. The mutations that appeared more than once were: m.5967T>C (unreported); m.6580G>A (unreported); m.6798G>A (unreported); m.6931G>A (unreported); m.7028C>T (unreported); m.7258T>A (rs1556423260); m.7275T>C (rs267606884); m.7312T>C (unreported); m.7403A>C (rs386829006); m.7935T>C (rs1603221222); m.9477-T (unreported) (the only one that appeared three times in the DB); and m.9645G>A (unreported).

**Figure 1:**
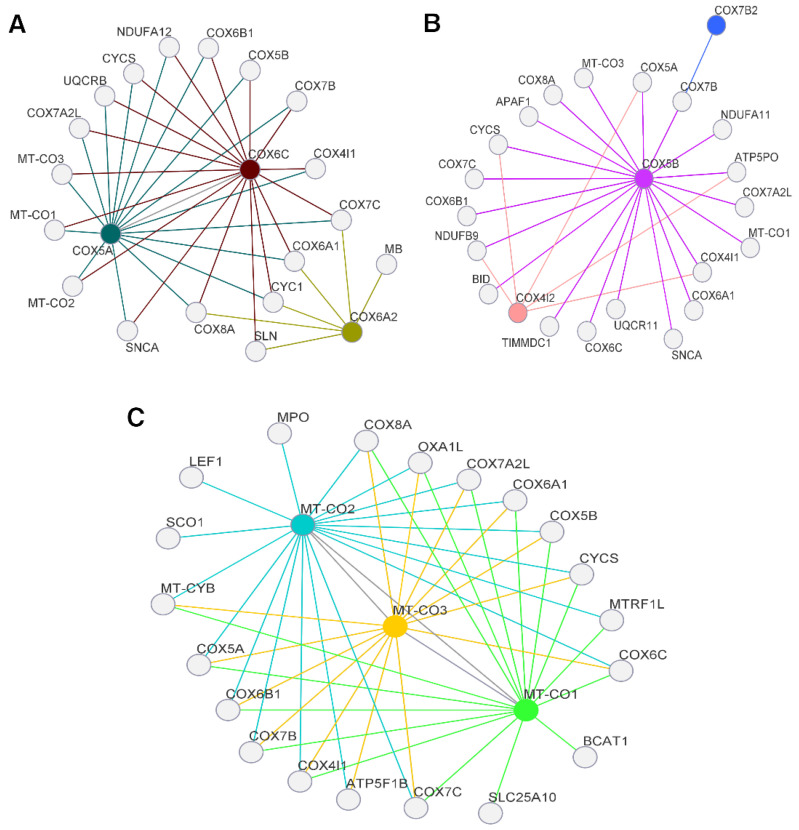
Interactions between the genes with the highest number of alterations and those that had variants with high pathogenicity. The differences in colors indicate the main gene (hub gene) and the peripheral genes with which it has a close physical association, and that may be associated with cancerous genetic pathway process. (**A**) Nuclear genes with pathogenic variants and their association with other genes, mostly related to apoptosis and OXPHOS. (**B**) Nuclear genes with the highest number of alterations. *COX5B* showed interaction with all the network genes, while *COX4I2* and *COX7B2* showed similar connections as the mitochondrial genes, in addition to the apoptotic cascade (*p* < 0.01), being a positive regulator of both extrinsic and intrinsic pathways, and epithelial tissue development, as well as to angiogenesis (*p* ≤ 0.01). (**C**) Mitochondrial genes participating in COX, from the systematization of KEGG and GO. Besides OXPHOS, these genes are involved in different processes (*p* < 0.01), such as muscle contraction (*p* < 0.01), apoptotic cascade (*p* < 0.01), as well as myopathies and other diseases like Alzheimer’s, Parkinson’s, and carcinogenesis due to the production of ROS. Furthermore, they are essentially associated with mitochondrial diseases such as Leber’s Hereditary Optic Neuropathy (LHON) and Kearns–Sayre Syndrome. Source: Adapted from GeneMANIA, 2022.

Variants rs199476129 (*MT-CO1*), rs199476128 (*MT-CO1*), rs267606884 (*MT-CO1*), rs267606611 (*MT-CO3*) and rs267606614 (*MT-CO3*) were the only reported, and, among them, rs199476128 and rs267606611 were described as benign, while the others have a pathogenic phenotypic impact, as seen in Supplementary Tables S1 and S3. Another variant with high impact but not yet reported in the literature is rs201617272 (*MT-CO1*). Until this work, none of the alterations found in this research have been reported in people with BC. As shown in Figure 1, the mtDNA genes (Figure 1C) have physical interactions with the COX protein and other OXPHOS structural genes, such as *MT-CYB*, *OXA1L*, *SCO1*, *ATP5F1B* and *CYCS*, as seen by a difference in colors for each connection for the main genes that were studied.

Regarding *MT-CO1*, the rs199476129 variant, first reported by [64], was shown to have a severe impact in an individual with myoglobinuria, with heteroplasmy (moderate morbidity), corroborating its prediction and suggesting it leads to instability of OXPHOS in muscle fibers, related to intolerance to continuous and long exercises. Variant rs199476128 was described first by [65] in an individual with Juvenile Myoclonic Epilepsy (JME) and early death. In addition, the variant was present in the mother and sister with JME (disease history), and it was difficult to report the pathogenicity of the alteration since another malignant mitochondrial variant (rs199474817) was present in the family. The rs199476129 variant has a clinically benign effect and has not been dated yet in any type of cancer, suggesting that it may not be a risk for BC.

Meanwhile, the rs267606884 variant, first reported by [66] in colorectal cancer (CRC), showed no relationship to tumor expansion. In another study [67], this variant was investigated in vitro and resulted in an increase in ROS, destabilization of OXPHOS and a decrease in the growth rate and expression of the structural COX, in line with our functional enrichment. Thus, they suggested an alteration with a high pathogenic degree, corroborating our result.

Additionally, the strong relation between genes like *CYCS* (Figure 1C) has been studied, but no strong relationship with *MT-CO1* has been found [68]. However, their research did find a relationship with anti-apoptotic genes; alterations in *MT-CO2* can lead to apoptosis by activating proteins such as α-synuclein (SNCA—Figure 1B), which is related to other genes, such as *COX5B* and *MT-CO3* [69]. The variants identified in *MT-CO2* were not dated until this work, but previous studies have demonstrated relations of the expression of this gene in nucleus–mitochondria interaction, presence in the vascular stroma, development of BC in people with European–American ethnicity and the knockdown of this gene, leading to a decrease in breast tumor growth in vitro [70,71,72,73].

For the *MT-CO3* gene, the rs267606611 variant, first reported by [74] in patients with Leber’s Hereditary Optic Neuropathy (LHON), suggests a possible association with human diseases. In 1994, [75] found this variant in individuals with optic neuropathy and peripheral neuropathy, showing a possible relationship to OXPHOS instability, mitochondrial activity and ocular dysfunction, characterizing it as a pathogenic variant. On the other hand, a study by [76] did not find the variant in patients with LHON and indicated that rs267606611 may not have an influence on the expression of COX in in vitro experiments, and there was no type of instability in the reduction in its kinetic activity, suggesting that the alteration has a benign phenotypic effect, corroborating our study.

The rs267606614 variant was first reported by [77] in a patient with Leigh’s Syndrome with a homoplasmic configuration. No type of abnormality was found in the mutant transcripts, but there was instability in the assembly of COX, suggesting *MT-CO3* as an intrinsic factor of organization in the assembly of COX. Studies by [78,79] observed other alterations in *MT-CO3* and *MT-CO1* in endometrial cancer and leukemia that do not represent a risk factor for the development of these types of cancer.

### 3.2. Presence of Alterations in Nuclear Genes

Forty-five variants were identified from the 17 nuclear genes investigated here, of which 31 alterations were not reported (considered new or somatic), and 14 are reported in the dbSNP. *COX5B* has the highest number of variants (n = 6), along with *COX4I2* (n = 5) and *COX7B2* (n = 5), which presented a high number of alterations, with no repeated alterations. Supplementary Table S2 shows all alterations found in nDNA. In addition, 37.8% alterations were missense (n = 17), 24.4% were silent/synonymous (n = 11), 15.5% in UTR 3’ site (n = 7), 13.3% were frameshift (n = 6), 4% were nonsense (n = 2), 2.2% were in the splicing site (n = 1) and 2.2% were in the UTR 5′ prime site (n = 1).

As for the genes with the highest number of alterations, no study was found relating *COX4I2* to any type of cancer, and there is no reported pathogenicity of its variants, so the present work was the first with this indication. In our cohort, two patients have the Luminal A molecular subtype, of which one deceased patient carried a variant in position 20:30227901G>A, as indicated in Supplementary Tables S2 and S3. In CRC, this gene was correlated with low survival and unfavorable prognosis, activation of tumor angiogenesis and fibroblast’s cytokines [80], and, in lung adenocarcinoma, it exhibited a relationship with apoptosis, in line with our findings (Figure 1B), indicating that *COX4I2* has a physical interaction with *CYCS* and that it could be used in early detection panels for cancer [81].

*COX5B* expression is significantly increased in BC when compared to cancer-free tissue, and it is associated with tumor size. The knockdown is related to a favorable prognosis, leading the tumor cells to cellular senescence and apoptosis, migration of neoplastic cells and decreased cell proliferation, converging with our results (Figure 1B), with direct interaction with pro-apoptotic genes *APAF1*, *CYCS* and *BID*, and it is the only one to interact with all COX subunits, consequently promoting cytokine secretion. This suggests the production of therapies that inhibit not only *COX5B* in target tumor cells but also their microenvironment. In addition, it causes increased ROS production and decreased mitochondrial potential, which can be seen as recurrently altered in patients with Luminal A and Basal-l molecular subtypes. Our results also reinforce the literature: out of the six patients with these mutations, three had Luminal A subtype, two Basal-l and one Luminal B [82,83].

Not yet associated to BC, the *COX7B2* gene is seen as a risk factor in the development of Hepatocellular Carcinoma (HCC) and is related to the proliferation, migration and invasion of liver cells [84]. Another study found a rare alteration in the *COX7B2* gene that has a high risk for the development of nasopharyngeal carcinoma [85]. *COX7B2* was one of the genes in the present work with the highest number of alterations. However, four of these mutations have not been previously reported, and one that has already been reported has shown no association with cancer until this study. Altered expression of *COX7* was seen as an opposite relationship to the Warburg Effect in people with BC [86].

Regarding genes with significantly impactful alterations present in nDNA, the SNPs rs780396486 (*COX5A*), rs769482258 (*COX6A2*) and rs753969142 (*COX6C*) were the main variants found in the nuclear genes (Supplementary Table S3). Regarding rs780396486, the patient has histological subtype Invasive Ductal Carcinoma (IDC) and molecular HER2, without distant metastasis (M0) and stage II; as for rs769482258, the patient had luminal A molecular subtype and stage III, non-specific histological subtype, MX metastasis; and, for the rs753969142, the patient exhibited histological type IDC, molecular HER2, stage III and M0.

In addition, the expression of *COX5A* is related to poor prognosis in patients with positive estrogen receptors (ER+) and chemotherapy resistance, exhibiting high histological invasion, larger tumor size and distant metastasis, leading to a more aggressive subtype Basal-l, being highly expressed and deregulated [87,88]. The knockdown of *COX5A* seems to cause the loss of COX affinity to oxygen and, consequently, mitochondrial dysfunction, which can lead to a protective effect, with inhibition of cancerous proliferation, migration and invasion in ER+ cells, suggesting that its decreased activity may help in the treatment. In the present study, variant rs780396486 suggests a possible factor for poor prognosis [88].

As for *COX6A2*, it appears to significantly influence the prognosis of people with HCC, and it is also associated with protection in people with esophageal cancer [89]. Variant rs769482258, found in another study, was significantly related to the development process of Parkinson’s disease. In this study, that variant has a high pathogenicity score; however, it has not yet been reported in any type of cancer, and it might be related to BC based on our results [90].

On the other hand, *COX6C* is seen as a hub gene to BC, with hypomethylation of the gene associated with this high expression, revealing that epigenetic factors of COX may be related. Furthermore, this gene is linked to the ER and can be associated to the study of new treatments, since this gene may influence therapeutic outcomes [91,92,93]. *COX6C* overexpression compensates for mitochondrial instability, decreasing ROS, which could lead to apoptosis in tumor cells. Here, we found variant rs753969142, with a high pathogenicity score, which could lead to the breakdown of the *COX6C* protein, being a potential protection factor to be analyzed and confirmed in future research [92].

### 3.3. Clinical Characterization of Nuclear Gene Databases

**Figure 2:**
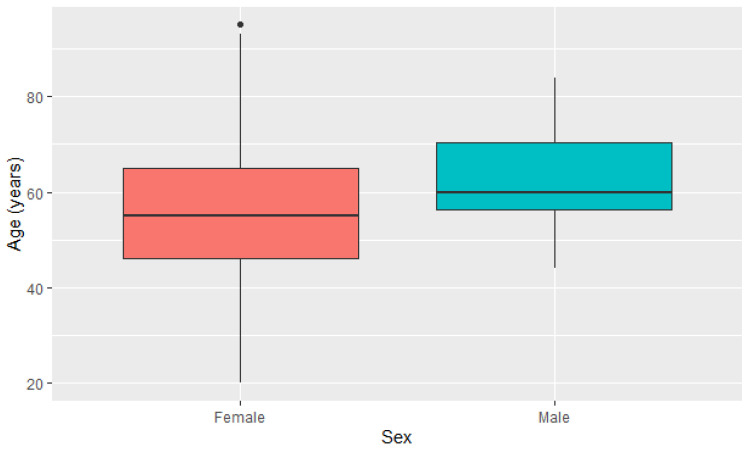
Diagram of age distribution in relation to the sex of the patients.

Regarding the clinical aspects in P2, the most affected people in our sample group were women (*p* < 0.001, Shapiro–Wilk test) in relation to men (Figure 2), in line with the literature, where women are more affected by conditions associated with human physiology, with even greater diversity in relation to age. Overall, 1991 individuals were identified, being 85.4% (n = 1701) female, 0.6% (n = 12) male and 13.9% (n = 278) had no reported data (ND, no data) in the DB where it is present. Thus, in addition to females being the most affected, which was already expected, they were also the group with the highest affected age range (20 to 95 years). Furthermore, the proportional number of females affected by alterations in the nuclear genes of COX was notably higher than males [94].

In general, it is possible to verify in Table 3 that there was no significant correlation in relation to the distribution of clinical characteristics in people who presented alterations in COX (with) and those who did not (without), which represents one of the main limitations of our study, since the merging of different DBs demonstrated an information gap or missing data in some DBs; therefore, we can only unify the most important information in common between these DBs. Furthermore, there are still no data that mention the relationship with clinical characteristics. A previous study indicated that mutations in *COX5B* can be seen as linked to BC, not yet related to a specific condition, and a study on *COX6A2* suggested this gene as an independent factor in the development of cancer [83,95].

**Figure 3:**
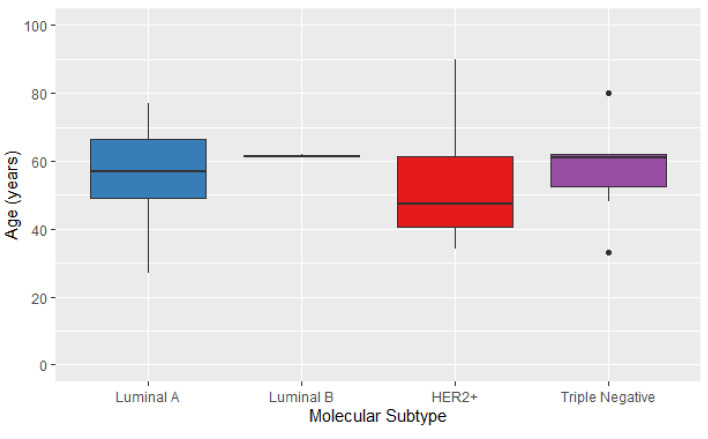
Distribution of molecular subtypes in the case group by age.

Based on this result, clinical data specifically from women with COX alterations were analyzed. These patients had significant differences (χ^2^, *p* = 0.007) in the distribution of molecular subtypes within the affected group (Figure 3). The molecular subtype Luminal A presented the highest frequency; HER2 presented the most diverse range, mainly affecting younger people; and molecular subtypes Luminal B and Basal-like (triple negative) appeared less frequently. This analysis is in line with the most common characteristics of our cohort and the literature, representing no difference from people with mutations in COX [3,96].

Furthermore, regarding the histological subtype of patients with COX alterations, women with BC presented mainly the IDC type (n = 14) (χ^2^, *p* = 0.042), followed by unspecified subtype (n = 4) and Invasive Lobular Carcinoma (ILC) (n = 7). Furthermore, among women with COX mutations, mostly stage II BC was found (χ^2^, *p* = 0.002), followed by stage III and IV. As for staging of metastasis, people in M0 (n = 18) were observed, with no sign of distant metastasis (χ^2^, *p* = 0.002), followed by M1 (n = 12) and MX (n = 2). Regarding these characteristics, they also do not present any difference between what is already shown in the literature and what is more common in our sample [97,98].

## 4. Conclusions

The mitochondrial and nuclear variants found here may be related to mitochondrial diseases, and none of these alterations, except for rs267606884, had been previously described in cancer. Furthermore, the characterized alterations (rs201617272, rs199476129, rs267606884, rs267606611, rs267606614, rs780396486, rs769482258 and rs753969142) should be investigated in different BC cohorts. Severe pathogenic effects of some of the found variants (rs267606614, rs267606884, rs753969142 and rs199476128) suggest these as potential biomarkers for BC. In our results, no significant difference in the clinical characteristics was found, suggesting that mutations in the structure of COX may act as independent factors.

Finally, these genes are seen as associated with tumor progression, possibly being biomarkers for BC and other types of cancer. This suggests that OXPHOS, and specifically COX, may be involved against the Warburg effect in BC. Nine genes (*MT-CO1*, *MT-CO2*, *MT-CO3*, *COX4I2*, *COX5A*, *COX5B*, *COX6A2*, *COX6C* and *COX7B2*) are recommended for investigation in future research in relation to cancer, especially when relating its expression and function in a cancer cell. Despite some limitations, such as missing data in the DBs, our study demonstrates new findings that may be linked to treatment, diagnosis and prognosis in BC. This is the first work that reports understudied COX mutations in patients with BC, reinforcing that OXPHOS and this type of cancer may be closely related.

## Figures and Tables

**Table 1:** Characterization of the investigated Complex IV genes.

Gene	Name	Protein	Locus	Reference
*Mitochondrially encoded cytochrome c oxidase I*	*MT-CO1*	COX1	m.5904-7445	[25]
*Mitochondrially encoded cytochrome c oxidase II*	*MT-CO2*	COX2	m.7586-8269	[25]
*Mitochondrially encoded cytochrome c oxidase III*	*MT-CO3*	COX3	m.9207-9990	[25]
*Cytochrome c oxidase subunit 4I1*	*COX4I1*	COX4	16q24.1	[26]
*Cytochrome c oxidase subunit 4I2*	*COX4I2*	COX4	20q11.21	[26]
*Cytochrome c oxidase subunit 5A*	*COX5A*	*COX5A*	15q24.2	[25]
*Cytochrome c oxidase subunit 5B*	*COX5B*	*COX5B*	2.11.2	[27]
*Cytochrome c oxidase subunit 6A1*	*COX6A1*	COX6A1 or COX6A	12q24.31	[27]
*Cytochrome c oxidase subunit 6A2*	*COX6A2*	COX6A2 or COX6A	16p11.2	[27]
*Cytochrome c oxidase subunit 6B1*	*COX6B1*	COX6B1 or COX6B	19q13.12	[25]
*Cytochrome c oxidase subunit 6B2*	*COX6B2*	COX6B2 or COX6B	19q13.42	[25]
*Cytochrome c oxidase subunit 6C*	*COX6C*	COX6C	8q22.2	[25]
*Cytochrome c oxidase subunit 7A1*	*COX7A1*	COX7A	19q13.12	[26]
*Cytochrome c oxidase subunit 7A2*	*COX7A2*	COX7A2 or COX7A	6q14.1	[26]
*Cytochrome c oxidase subunit 7B*	*COX7B*	COX7B	Xq21.1	[25]
*Cytochrome c oxidase subunit 7B2*	*COX7B2*	COX7B2 or COX7B	4p12	[25]
*Cytochrome c oxidase subunit 7C*	*COX7C*	COX7C	5q14.3	[25]
*Cytochrome c oxidase subunit 8A*	*COX8A*	COX8A or COX8	11q13.1	[26]
*Cytochrome c oxidase subunit 8C*	*COX8C*	COX8C or COX8	14q32.12	[26]
*Cytochrome c oxidase subunit fa4*	*NDUFA4*	NDUFA4 or COXFA4	7p21.3	[28]

**Table 2:** Characterization of the analyzed mtDNA and nDNA databases of breast cancer samples.

Material	Database	Samples	Reference
mtDNA	The Cancer Mitochondria Atlas	216	[32]
nDNA	Breast Invasive Carcinoma (British Columbia, Nature 2012)	65	[33]
	Breast Invasive Carcinoma (Broad, Nature 2012)	103	[34]
	Breast Invasive Carcinoma (Sanger, Nature 2012)	100	[35]
	Metastatic Breast Cancer (INSERM, PLoS Med 2016)	216	[36]
	Breast Invasive Carcinoma (TCGA, PanCancer Atlas)	1084	[37,38,39,40,41,42,43,44,45,46,47]
	The Metastatic Breast Cancer Project (Provisional, December 2021)	301	[48,49,50]
	Proteogenomic landscape of breast cancer (CPTAC, Cell 2020)	122	[51]

**Table 3:** Characteristics of BC patients found in cBioPortal, frequency ratio of patients and non-carriers of COX alterations.

Age by Sex			
	Median	Minimum	Maximum
**Female (n = 1701)**	55.9 ± 13.694	20.0	95.0
**Male (n = 278)**	62.6 ± 12.391	44.0	84.0
**Frequency Distribution**			
**Alteration**	**Sex**	**Frequency (%)**	**χ** **^2^**
With	Female	28 (75.7)	*p =* 0.654
	Male	0 (0.0)	
	ND	9 (24.3)	
Without	Female	1673 (85.6)	
	Male	12 (0.6)	
	ND	269 (13.8)	
**Alteration**	**Molecular Subtype**	**Frequency (%)**	**χ** **^2^**
With	Normal-like	0 (0.0)	*p* = 0.108
	Luminal A	14 (37.8)	
	Luminal B	2 (5.4)	
	HER2	4 (10.8)	
	Basal-like	8 (21.6)	
	ND	9 (24.4)	
Without	Normal-like	75 (3.8)	
	Luminal A	804 (41.2)	
	Luminal B	337 (17.3)	
	HER2	114 (5.8)	
	Basal-like	288 (14.7)	
	ND	336 (17.2)	
**Alteration**	**Cancer staging**	**Frequency (%)**	**χ** **^2^**
With	I	5 (13.5)	*p =* 0.714
	II	15 (40.5)	
	III	5 (13.5)	
	IV	2 (5.4)	
	ND	10 (27.1)	
Without	I	235 (12.1)	
	II	840 (42.9)	
	III	448 (22.9)	
	IV	87 (4.5)	
	ND	344 (17.6)	
**Alteration**	**Metastasis staging**	**Frequency (%)**	**χ** **^2^**
With	MX	2 (5.4)	*p =* 0.535
	M0	18 (48.7)	
	CM0 (I+)	0 (0.0)	
	M1	12 (32.4)	
	ND	5 (13.5)	
Without	MX	160 (8.2)	
	M0	1013 (51.8)	
	CM0 (I+)	6 (0.4)	
	M1	427 (21.8)	
	ND	348 (17.8)	

ND = No Data.

## Data Availability

The clinical data analyzed here, in addition to being currently available at cBioPortal (https://www.cbioportal.org/, accessed on 29 July 2022), were grouped and are available at https://doi.org/10.6084/m9.figshare.23260352, accessed on 29 July 2022.

## Supplementary Materials

The following supporting information can be downloaded at: https://www.mdpi.com/article/10.3390/genes14071465/s1, Table S1: Alterations found in mitochondrial DNA, displaying their location and the existence of reporting in dbSNP or global literature, Table S2: Characterization of alterations found in COX nuclear genes, Table S3: Characterization of the phenotypic impact of alterations reported in dbSNP on different predictors of pathogenicity.
