# Supplementary material for: Mutations in Structural Genes of the Mitochondrial Complex IV May Influence Breast Cancer
Source: Genes (Basel). 2023 Jul 18;14(7):1465. doi: 10.3390/genes14071465 (PMC10379055; doi:10.3390/genes14071465)
Supplement: Supplementary file 1 [file genes-14-01465-s001.zip › genes-2478996-supplementary.pdf]

SUPPLEMENTARY MATERIAL

Supplementary Table S1 – Alterations found in mitochondrial DNA, displaying their location and the existence of reporting in dbSNP or global literature.

Gene	Type	Locus	dbSNP	Reference
	nsSNP	m.5913G>A	rs201617272	-
	nsSNP	m.5917G>A	-	-
	nsSNP	m.5920G>A	rs199476129	[64]
	synSNP	m.5945C>T	rs1603220196	-
	nsSNP	m.5950G>A	-	-
	nsSNP	m.5967T>C*	-	-
	nsSNP	m.5997G>A	-	-
	synSNP	m.6023G>A	rs1603220229	-
	nsSNP	m.6028G>A	rs28580752	-
	nsSNP	m.6057C>T	-	-
	nsSNP	m.6061T>C	rs1603220252	-
	nsSNP	m.6102T>C	-	-
	nsSNP	m.6111G>A	-	-
	nsSNP	m.6117C>T	-	-
	nsSNP	m.6174G>A	rs1603220303	-
	nsSNP	m.6193T>C	-	-
	nsSNP	m.6202T>C	-	-
MT-CO1	Frameshift Ins	m.6218+C	-	-
	nsSNP	m.6225T>C	rs1603220329	-
	synSNP	m.6227T>C	rs1556423092	-
	nsSNP	m.6235T>A	-	-
	nsSNP	m.6253T>C	rs200165736	-
	nsSNP	m.6258G>C	-	-
	synSNP	m.6260G>A	rs201395766	[99]
	synSNP	m.6305G>A	rs879141792	-
	nsSNP	m.6337T>C	rs1603220425	-
	nsSNP	m.6346T>C	-	-
	nsSNP	m.6358T>C	rs1603220439	-
	nsSNP	m.6457T>C	-	-
	nsSNP	m.6480G>A	rs199476128	[65]
	nsSNP	m.6481T>C	rs28721398	-
	nsSNP	m.6513G>C	-	-
	nsSNP	m.6580G>A*	-	-
	synSNP	m.6620T>C	-	-
	nsSNP	m.6637T>C	-	-

	nsSNP	m.6646T>C	-	-
	nsSNP	m.6673T>C	-	-
	nsSNP	m.6684T>C	-	-
	nsSNP	m.6723G>A	rs1603220595	-
	nsSNP	m.6724T>C	rs28393263	-
	nsSNP	m.6760T>C	-	-
	synSNP	m.6776T>C	rs41473545	-
	nsSNP	m.6798G>A*	-	-
	nsSNP	m.6822T>C	rs1603220640	-
	nsSNP	m.6825G>A	rs1603220642	-
	nsSNP	m.6838T>C	-	-
	nsSNP	m.6931G>A*	-	-
	nsSNP	m.6933T>C	rs1603220698	-
	nsSNP	m.6937T>C	rs1603220701	-
	nsSNP	m.6976T>C	-	-
	nsSNP	m.7008G>A	-	-
	synSNP	m.7022T>C	rs879059323	-
	synSNP	m.7028C>T*	-	-
	nsSNP	m.7032T>C	rs1603220739	-
	nsSNP	m.7045T>C	-	-
	synSNP	m.7049A>G	rs1603220745	-
	nsSNP	m.7056G>A	rs1556423224	-
	nsSNP	m.7074G>A	-	-
	nsSNP	m.7078G>A	-	-
	nsSNP	m.7092T>C	-	-
	nsSNP	m.7119G>A	rs1556423235	-
	nsSNP	m.7142T>G	-	-
	synSNP	m.7151C>T	rs1603220804	-
	nsSNP	m.7153T>G	-	-
	nsSNP	m.7191T>C	rs1603220824	-
	nsSNP	m.7201T>C	-	-
	nsSNP	m.7219G>A	-	-
	nsSNP	m.7242T>C	-	-
	nsSNP	m.7258T>C*	rs1556423260	-
	synSNP	m.7268T>C	-	-
	nsSNP	m.7275T>C*	rs267606884	[66, 67]
	nsSNP	m.7312T>C*	-	-
	synSNP	m.7325A>G	rs1556423269	-
	nsSNP	m.7332G>C	rs1603220889	-

	synSNP	m.7334T>C	rs2068702871	-
	Frameshift Ins	m.7396+CCCCCC	rs1603220944	-
	synSNP	m.7403A>C*	rs386829006	-
MT-CO2	nsSNP	m.7595G>A	-	-
	synSNP	m.7609T>C	rs1603221036	-
	nsSNP	m.7652T>C	-	-
	nsSNP	m.7658G>A	-	-
	nsSNP	m.7778T>C	-	-
	nsSNP	m.7803T>C	-	-
	nsSNP	m.7809T>C	-	-
	synSNP	m.7849C>T	rs386829015	-
	nsSNP	m.7869T>C	-	-
	nsSNP	m.7902G>A	-	-
	nsSNP	m.7935T>C*	rs1603221222	-
	nsSNP	m.7936C>A	-	-
	nsSNP	m.7976G>A	rs377368526	-
	nsSNP	m.7977G>A	-	-
	nsSNP	m.7986G>A	-	-
	nsSNP	m.8027G>A	rs1116904	-
	nsSNP	m.8072T>C	-	-
	nsSNP	m.8088T>C	-	-
	nsSNP	m.8153G>A	-	-
	nsSNP	m.8213G>A	-	-
	nsSNP	m.8219G>A	-	-
	nsSNP	m.8225A>G	rs1603221335	-
	nsSNP	m.8243G>A	-	-
	nsSNP	m.8249G>A	-	-
MT-CO3	nsSNP	m.9264G>A	-	-
	synSNP	m.9266G>A	rs374335946	-
	nsSNP	m.9267G>A	rs1556423650	-
	nsSNP	m.9285A>T	-	-
	nsSNP	m.9326A>T	-	-
	Frameshift Ins	m.9335+AT	-	-
	nsSNP	m.9438G>A	rs267606611	[74, 75, 76]
	Frameshift Del	m.9477-T**	-	-
	synSNP	m.9494A>G	rs1556423680	-
	nsSNP	m.9500C>A	-	-
	Frameshift Ins	m.9531+CCCCCC	rs267606614	[77]

	nsSNP	m.9643G>A	-	-
	nsSNP	m.9645G>A*	-	-
	nsSNP	m.9658T>C	-	-
	nsSNP	m.9720T>C	-	-
	nsSNP	m.9753G>A	rs1569484322	-
	nsSNP	m.9832T>C	-	-
	nsSNP	m.9850T>C	rs1603222543	-
	nsSNP	m.9906G>A	-	-
	nsSNP	m.9912G>A	rs28580363	-
	nsSNP	m.9918G>C	-	-
	nsSNP	m.9979G>A	rs1603222604	-
	nsSNP	m.9985G>A	-	-

nsSNP = non-synonymous single nucleotide polymorphisms; synSNP = synonymous single nucleotide polymorphisms; Frameshift Ins = Frameshift Insertion; Frameshift Del = Frameshift Deletion.

- = not reported in the literature (Unreported).

** One repetition of the change (n=2)*

*** Two repetitions of the change (n=3)*

Supplementary Table S2 – Characterization of alterations found in COX nuclear genes.

Gene	Alteration	Locus	Type	Impact	dbSNP
COX4I1	+T	16:85839347	*Frameshift Ins*	High	-
	C>T	16:85840354	*Silent*	Low	-
	C>T	16:85840355	*Missense*	Moderate	-
COX4I2	G>A	20:30227749	*Silent*	Low	rs1050846788
	G>A	20:30227833	*Silent*	Low	-
	A>G	20:30227892	*Missense*	Moderate	-
	G>A	20:30227901	*Splice site*	High	-
	C>A	20:30232760	*UTR 3' prime*	Modifier	rs774033084
COX5A	G>A	15:75221511	*Missense*	Moderate	rs780396486
COX5B	G>C	2:98262600	*Silent*	Low	-
	C>A	2:98262609	*Silent*	Low	-
	G>A	2:98262630	*Silent*	Low	-
	G>T	2:98263556	*Missense*	Moderate	-
	A>G	2:98264501	*Missense*	Moderate	-
	G>A	2:98264516	*Missense*	Moderate	rs746587601
COX6A1	G>A	12:120876255	*Silent*	Low	rs1463183756
	C>T	12:120875960	*Missense*	Moderate	-
	C>G	12:120876217	*Missense*	Moderate	-
COX6A2	-C	31439163	*Frameshift Del*	High	rs769482258
	C>T	31439585	*Missense*	Moderate	rs2082154234
COX6B1	C>T	19:36145521	*Missense*	Moderate	rs1451939305
	-C	19:36145554	*Frameshift Del*	High	-
COX6B2	G>A	19:55865104	*Silent*	Baixa	-
COX6C	G>A	8:100899731	*UTR 3' prime*	Modifier	-
	C>A	8:100899792	*Missense*	Moderate	rs753969142
COX7A1	G>C	19:36642577	*Silent*	Low	-
	-C	19:36642366	*Frameshift Del*	High	-
COX7A2	C>T	6:75947625	*UTR 3' prime*	Modifier	rs773507804
	A>G	6:75947696	*Missense*	Moderate	-
	T>C	6:75950100	*Missense*	Moderate	rs771323246
COX7B	-T	X:77158195	*Frameshift Del*	High	-
	G>C	X:77160724	*Missense*	Moderate	-
COX7B2	A>C	4:46736933	*UTR 3' prime*	Modifier	-
	C>A	4:46736955	*UTR 3' prime*	Modifier	-
	G>C	4:46736959	*UTR 3' prime*	Modifier	rs771248119
	C>A	4:46736981	*Nonsense*	High	-
	G>A	4:46737164	*Nonsense*	High	-
COX7C	G>C	5:85913930	*Missense*	Moderate	-
	G>A	5:85915272	*Missense*	Moderate	-
	G>C	5:85915321	*UTR 3' prime*	Modifier	rs1476761587
COX8A	C>A	11:63742117	*UTR 5' prime*	Modifier	-

	C>T	11:63742177	*Silent*	Baixa	-
	A>T	11:63743764	*Missense*	Moderate	-
COX8C	C>T	14:93813698	*Silent*	Baixa	rs1389396955
NDUFA4	+G	7:10978513	*Frameshift Ins*	High	-

Ins, Insertion; Del, Deletion.

Supplementary Table S3 – Characterization of the phenotypic impact of alterations reported in dbSNP on different predictors of pathogenicity.

Variant	Position	Phenotypic Impact		
		PolyPhen	***ClinVar***	***SIFT***
rs201617272	m.5913G>A	-	*Pathogenic*	-
rs199476129	m.5920G>A	-	*Pathogenic*	-
rs28580752	m.6028G>A	-	-	-
rs1603220252	m.6061T>C	-	*Uncertain significance*	-
rs1603220303	m.6174G>A	-	*Uncertain significance*	-
rs1603220329	m.6225T>C	-	-	-
rs200165736	m.6253T>C	-	*Benign*	-
rs1603220425	m.6337T>C	-	-	-
rs1603220439	m.6358T>C	-	*Uncertain significance*	-
rs199476128	m.6480G>A	-	*Benign*	-
rs28721398	m.6481T>C	-	*Uncertain significance*	-
rs1603220595	m.6723G>A	-	*Benign*	-
rs28393263	m.6724T>A	-	-	-
rs1603220640	m.6822T>C	-	-	-
rs1603220642	m.6825G>A	-	-	-
rs1603220698	m.6933T>C	-	-	-
rs1603220701	m.6937T>C	-	-	-
rs1603220739	m.7032T>C	-	*Uncertain significance*	-
rs1556423224	m.7056G>A	-	-	-
rs1556423235	m.7119G>A	-	*Benign*	-
rs1603220824	m.7191T>C	-	*Likely benign*	-
rs1556423260	m.7258T>C	-	*Benign*	-
rs267606884	m.7275T>C	-	*Pathogenic*	-
rs1603220889	m.7332G>A	-	*Uncertain significance*	-
rs1603220944	m.7396+CCCCCCC	-	-	-
rs1603221222	m.7934T>C	-	-	-
rs377368526	m.7976G>A	-	*Uncertain significance*	-
rs1116904	m.8027G>A	-	*Benign*	-
rs1603221335	m.8225A>G	-	*Uncertain significance*	-
rs1556423650	m.9267G>A	-	*Uncertain significance*	-
rs267606611	m.9438G>A	-	*Benign/ Pathogenic*	-
rs267606614	m.9537+CCCCCCC	-	*Pathogenic*	-
rs1569484322	m.9752G>A/C	-	-	-
rs1603222543	m.9849T>C	-	-	-
rs28580363	m.9911G>A	-	-	-
rs1603222604	m.9979G>A	-	-	-
rs774033084	chr20:30232760	-	-	-
rs780396486	chr15:74929170	*Probably Damaging (s = 0.967)*	-	*Deleterious (s = 0.010)*
rs746587601	chr2:98264516	*Benign (s = 0.319)*	-	*Tolerated (s = 0.070)*
rs769482258	chr16:31439163	*Probably Damaging (s = 0.947)*	-	*Deleterious (s = 0.000)*

rs2082154234	chr16:31439584	-	-	-
rs1451939305	chr19:36145521	*Benign (s = 0.049)*	-	*Deleterious (s = 0.030)*
rs753969142	chr8:100899792	*Possibly Damaging (s = 0.665)*	-	*Tolerated (s = 0.00)*
rs773507804	chr6:75947625	-	-	*Deleterious - Low Confidence (s = 0.020)*
rs771323246	chr6:75950100	*Benign (s = 0.139)*	-	*Deleterious (s = 0.000)*
rs771248119	chr4:46736959	-	-	-

S: *score or level of pathogenicity.*

- : *Unreported.*
